# Clinical and Epidemiological Profile of Progressive Supranuclear Palsy in a Colombian Referral Center: A Retrospective Cohort Study

**DOI:** 10.1155/nri/9076106

**Published:** 2026-10-29

**Authors:** Marcela Henao-Pérez, Diana Carolina López-Medina, Alejandra Arboleda-Ramírez, Zahian Barroso-Barrera, Juan Pablo Londoño-Aristizábal

**Affiliations:** ^1^ Universidad Cooperativa de Colombia Medellín Colombia ucc.edu.co; ^2^ Hospital Neurológico de Colombia Medellín Colombia

**Keywords:** atypical parkinsonism, Colombia, epidemiology, phenotype, progressive supranuclear palsy

## Abstract

**Background:**

Progressive supranuclear palsy (PSP) is a rare 4‐repeat tauopathy characterized by marked clinical heterogeneity and frequent diagnostic delay. Epidemiological and phenotypic data from Latin America remain limited. This study aimed to describe the clinical and epidemiological characteristics of PSP in a Colombian referral center and to compare findings by sex and phenotype.

**Methods:**

We retrospectively reviewed PSP cases evaluated at a Colombian neurological referral center from 2013 to 2023. Demographic, clinical, neuroimaging, diagnostic, and treatment‐related variables were extracted from medical records. PSP phenotypes were retrospectively classified and grouped using clinical information from medical records. Period prevalence was estimated using the adult population of Antioquia as the denominator.

**Results:**

Among 152 patients, 53.9% were male. Crude period prevalence was 1.8 per 100,000 adults, peaking at 75–84 years. Women were older than men at evaluation, whereas age at symptom onset was similar between sexes. Mean diagnostic delay was approximately 3.5 years. Postural instability was the most frequent presenting symptom. PSP‐Richardson syndrome and PSP‐parkinsonism were the most common phenotypes. MRI was the most frequently requested imaging study, and midbrain atrophy was the most common abnormality. Levodopa/carbidopa was the most frequently prescribed medication, with documented initial response in only a subset of treated patients.

**Conclusions:**

PSP in this Colombian cohort predominantly affected older adults and was characterized by substantial diagnostic delay, marked phenotypic heterogeneity, and predominantly symptomatic treatment. These findings expand the limited Latin American literature and underscore the need for earlier recognition of both typical and atypical PSP presentations.

## 1. Introduction

Progressive supranuclear palsy (PSP), also known as Steele–Richardson–Olszewski syndrome, was first described in 1964 in a neuropathological report based on nine cases [1]. It is an uncommon neurodegenerative disorder, a predominantly subcortical 4‐repeat tauopathy associated with aging, and one of the main atypical parkinsonian syndromes considered in the differential diagnosis of Parkinson’s disease (PD) [2, 3].

The mean age at symptom onset is approximately 63 years, and onset before the age of 40 years is exceptional. Reported prevalence estimates range from 1.4 to 6.4 cases per 100,000 inhabitants [4, 5]. Mean survival after symptom onset has been estimated at approximately 6.9 years [6]. Regarding sex distribution, some studies have suggested a slight male predominance [7], whereas a systematic review of prevalence studies found no clear sex differences in population‐based PSP rates [8]. Similarly, no consistent geographic, ethnic, or racial differences in PSP frequency have been established [9].

Several studies have suggested that PSP may be influenced by environmental and lifestyle‐related factors. Proposed risk factors include dietary exposure to Annonaceae, certain environmental toxins, frequent meat consumption, and chronic hypertension, whereas higher educational attainment, statin use, and estrogen therapy in women have been suggested as potentially protective. However, these findings derive mainly from observational studies and require further validation [10].

The characteristic clinical pattern of PSP and its variants includes vertical supranuclear gaze palsy, postural instability (PI) with backward falls during the first year after symptom onset, akinesia, and cognitive dysfunction [11]. However, PSP is clinically heterogeneous. To account for this variability, the Movement Disorder Society (MDS) published revised diagnostic criteria in 2017 that recognize several clinical subtypes, including PSP‐Richardson syndrome (PSP‐RS), PSP‐parkinsonism (PSP‐P), PSP with corticobasal syndrome (PSP‐CBS), PSP with progressive gait freezing (PSP‐PGF), PSP with predominant oculomotor dysfunction (PSP‐OM), PSP with predominant postural instability (PSP‐PI), PSP with predominant frontal presentation (PSP‐F), PSP with predominant speech/language disorder (PSP‐SL), PSP with predominant cerebellar ataxia (PSP‐C), and PSP with predominant primary lateral sclerosis (PSP‐PLS). This phenotypic framework has improved clinical characterization and is relevant for differential diagnosis and patient stratification in research settings [2, 12].

The diagnosis of PSP remains primarily clinical and is based on MDS criteria, with structural and functional neuroimaging serving mainly as supportive tools in the differential diagnosis, whereas definitive confirmation remains neuropathological at postmortem examination. To date, no disease‐specific biomarkers or disease‐modifying therapies are available. Therefore, treatment remains symptomatic and multidisciplinary, targeting motor symptoms, dysphagia, behavioral disturbances, and prevention of complications, with the aim of preserving function and quality of life [13, 14].

In Colombia, published evidence on PSP is scarce and largely limited to isolated case reports and very small series, with no systematic clinical descriptions. Accordingly, this study aimed to describe the clinical and epidemiological characteristics of patients with PSP diagnosed between 2013 and 2023 in Antioquia, Colombia, and to explore associations between sociodemographic and clinical variables in this cohort.

## 2. Methods

### 2.1. Study Design and Participants

We conducted a retrospective, observational, cross‐sectional study of patients with a confirmed clinical diagnosis of PSP. The study was based on a census of all PSP cases evaluated at a neurological referral center, between 2013 and 2023. All patients who met clinical criteria for PSP were included, with no exclusions based on sex, age, or other demographic or clinical characteristics.

### 2.2. Data Collection and Variables

Data were collected retrospectively through medical record review. Extracted variables included sociodemographic characteristics (age, sex, geographic origin, and type of health insurance), clinical features (PSP phenotype, presenting symptom, and time from symptom onset to diagnosis), personal medical history and comorbidities, relevant ancillary findings, including brain neuroimaging results, and treatment‐related variables, with particular emphasis on levodopa response as documented during clinical follow‐up.

Alternative diagnoses were identified from the diagnostic impressions, previous diagnoses, and differential diagnoses documented in the medical records. Their exclusion was based on neurological assessment at the referral center, application of clinical diagnostic criteria, longitudinal clinical follow‐up when available, and ancillary testing performed according to clinical suspicion. Ancillary studies included structural neuroimaging, VDRL testing, cerebrospinal fluid (CSF) analysis, transcranial sonography (TCS), computed tomography, magnetic resonance imaging, and magnetic resonance angiography, as applicable. Genetic testing and autoimmune antibody testing were not systematically performed in this retrospective cohort and were, therefore, not included as standardized variables.

Patients initially diagnosed with PD and later reclassified as PSP were identified when a prior diagnosis of PD was explicitly documented in the medical record before the final clinical diagnosis of PSP.

Levodopa exposure was retrospectively reconstructed from levodopa/carbidopa prescriptions documented during clinical follow‐up. The daily levodopa dose (mg/day) was calculated by multiplying the dose per administration by the dosing frequency; the carbidopa component was not included in this calculation. For each patient, we recorded the initial dose, maximum dose documented during follow‐up, and final recorded dose, when available. Maximum and final doses were analyzed separately because some patients underwent dose reductions after reaching higher levodopa doses.

Documented levodopa exposure was defined as the number of months between the first and last recorded prescriptions. This measure represents the treatment period captured in the medical record and does not confirm continuous treatment use or adherence. Initial therapeutic response to levodopa was retrospectively assessed from the treating neurologist’s documentation in the medical record. Patients with ambiguous or unverifiable dosing information were excluded from the quantitative dose analysis.

For analytical purposes, some variables were recategorized to facilitate comparisons. PSP phenotypes were grouped into two main categories: typical PSP, corresponding to PSP‐RS, and atypical PSP, which included all remaining phenotypes [15]. In addition, selected continuous variables were categorized when appropriate, such as age grouped by decade of life.

### 2.3. Prevalence Estimate

PSP prevalence was estimated as a period prevalence, considering all cases identified during the 2013–2023 study interval. The numerator was the total number of patients with PSP identified during this period, and the denominator was the population of Antioquia aged > 20 years according to the 2018 census of the National Administrative Department of Statistics (Departamento Administrativo Nacional de Estadística, DANE) [16]. Based on these data, a crude period prevalence rate was calculated for the adult population of the department. Age‐adjusted prevalence was subsequently estimated using direct standardization.

### 2.4. Statistical Analysis

Descriptive analyses were performed using absolute and relative frequencies. Relative frequencies (%) were calculated using the number of individuals in the corresponding subgroup as the denominator, according to sex or PSP phenotype. Hypothesis tests were used to assess differences between men and women and between PSP subtypes. All analyses were performed using Jamovi Version 2.6.44, and statistical significance was set at *p* < 0.05.

An exploratory binary logistic regression analysis was planned to assess associations between typical versus atypical PSP phenotypes and the clinical and sociodemographic variables included in the study. However, because of collinearity among predictors and substantial case loss related to missing data during principal component score generation, the final regression models were not estimable and were therefore not included in the final analysis [17, 18].

### 2.5. Ethical Considerations

The study protocol was approved by the neurological referral center Ethics Committee. In accordance with Colombian regulations (Resolution 8430 of 1993 issued by the Ministry of Health), the study was classified as risk‐free research because of its observational and retrospective design based on medical record review. Accordingly, the requirement for informed consent was waived. All data were handled under strict confidentiality, and personal information was anonymized to protect patient privacy. The study was conducted in accordance with the Declaration of Helsinki and the Belmont Report. The project also received technical‐scientific and financial approval from Universidad Cooperativa de Colombia under code INV 3727.

### 2.6. Use of Artificial Intelligence Tools

Artificial intelligence (AI) tools were used during manuscript preparation to assist with language editing and improvement of sentence structure and readability. AI tools were not used to generate original data, perform data analysis, interpret findings independently, or make scientific decisions. All text produced or modified with AI assistance was critically reviewed and revised by the authors. The authors verified factual accuracy, checked references, corrected errors, and ensured that the final content accurately reflected the study data and the authors’ scientific interpretation.

## 3. Results

A total of 152 patients with PSP evaluated between 2013 and 2023 were included in the analysis. Of these, 82 (53.9%) were men, most patients resided in the Metropolitan Area of Valle de Aburrá (80.9%), and approximately half were affiliated with the contributory health insurance system.

The crude period prevalence of PSP in Antioquia during 2013–2023 was estimated at approximately 1.8 cases per 100,000 adults aged ≥ 20 years, based on 152 identified cases. When stratified by sex, crude prevalence was slightly higher in men than in women (2.04 vs. 1.52 per 100,000). Age‐ and sex‐specific prevalence increased with age in both sexes and peaked in the 75‐ to 84‐year age group, reaching 0.96 per 100,000 in men and 0.72 per 100,000 in women. In individuals aged ≥ 95 years, prevalence was higher in women than in men (0.08 vs. 0.03 per 100,000), whereas prevalence was null or very low in those younger than 55 years (Table 1).

**TABLE 1:** Age‐ and sex‐specific period prevalence of progressive supranuclear palsy in Antioquia, Colombia, 2013–2023, with age‐adjusted overall prevalence estimates.

Age group (years)	Male cases	Female cases	Male population in Antioquia^∗^	Female population in Antioquia^∗^	Age‐specific prevalence in males per 100,000	Age‐specific prevalence in females per 100,000
< 55	1	0	2,973,868	3,203,838	0.02	0.00
55–64	7	5	550,732	672,178	0.18	0.11
65–74	27	11	308,840	441,208	0.76	0.22
75–84	35	36	143,602	195,764	0.96	0.72
85–94	11	14	43,566	69,934	0.33	0.26
≥ 95	1	4	5000	8018	0.03	0.08

Total	**82**	**70**	**4,025,608**	**4,590,940**	**2.04**	**1.52**

*Note:*Bold formatting was used solely to distinguish the total row from the age‐specific categories and does not indicate statistical significance.

^∗^Based on the 2018 population census of the National Administrative Department of Statistics (Departamento Administrativo Nacional de Estadística [DANE]) for the Department of Antioquia, Colombia [16].

Table 2 summarizes the sociodemographic and clinical characteristics according to sex. Seventy patients (46.0%) were women and 82 (54.0%) were men. Patients in both groups were older at the time of evaluation, with a mean age of 80.1 ± 9.26 years in women and 75.2 ± 10.0 years in men (*p* = 0.002). Most patients were in the 75‐ to 84‐year age range, and only a small proportion were older than 95 years (5.7% of women and 1.2% of men). Around 80% of patients resided in the Metropolitan Area of Valle de Aburrá, with no significant sex‐related differences. Health insurance status was similarly distributed between sexes, with the contributory and subsidized systems accounting for most cases.

**TABLE 2:** Sociodemographic and clinical characteristics according to sex.

Variable	Women (*n* = 70) *n* (%)	Men (*n* = 82) *n* (%)	*p* value
Age, years^a^	80.1 ± 9.26 [80 (7.75)]	75.2 ± 10.0 [76 (12.8)]	0.002
Age group, years			0.077^b^
< 55	0	1 (1.2)	
55–64	5 (7.1)	7 (8.5)	
65–74	11 (15.7)	27 (32.9)	
75–84	36 (51.4)	35 (42.7)	
85–94	14 (20.0)	11 (13.4)	
≥ 95	4 (5.7)	1 (1.2)	
Residence			0.69^b^
Metropolitan Area of Valle de Aburrá	55 (78.6)	68 (82.9)	
Outside the Metropolitan Area of Valle de Aburrá	13 (18.6)	13 (15.9)	
Health insurance			0.67^b^
Subsidized	27 (38.6)	31 (37.8)	
Contributory	37 (52.9)	40 (48.8)	
Special insurance scheme	3 (4.3)	2 (2.4)	
Private health insurance/prepaid medicine	0	1 (1.2)	
Self‐pay	2 (2.9)	7 (8.5)	
Family history			0.75^b^
Alzheimer’s disease	2 (2.9)	3 (3.7)	
Parkinson’s disease	2 (2.9)	3 (3.7)	
Alzheimer’s disease/Parkinson’s disease	1 (1.4)	0	
Dementia	2 (2.9)	3 (3.7)	
Olivopontocerebellar atrophy	0	1 (1.2)	
Personal medical history			
Hypertension	40 (57.1)	36 (43.9)	0.26^c^
Type 2 diabetes mellitus	15 (21.4)	15 (18.3)	0.72^c^
Hypothyroidism	21 (30.0)	10 (12.2)	0.024^c^
Dyslipidemia	11 (15.7)	11 (13.4)	0.80^c^
> 3 cardiovascular comorbidities	10 (14.3)	11 (13.4)	0.80^c^
Psychoaffective disorders	10 (14.3)	10 (12.2)	0.84^c^
Sleep disorders	2 (2.9)	6 (7.3)	0.36^b^
Age at symptom onset, years^a^	71.5 ± 9.41 [71 (12.8)]	69.6 ± 8.32 [67 (12.0)]	—
Presenting symptom			0.30^b^
Postural instability	25 (35.7)	30 (36.6)	
Akinesia or bradykinesia	15 (21.4)	25 (30.5)	
Cognitive dysfunction	20 (28.6)	13 (15.9)	
Oculomotor dysfunction	2 (2.9)	4 (4.9)	
Other	8 (11.4)	8 (9.8)	
Time from symptom onset to diagnosis, years^a^	3.98 ± 1.96 [4 (2)]	3.06 ± 2.32 [2 (3)]	0.002^d^
Clinical phenotype			0.75^b^
PSP‐RS	23 (32.9)	27 (32.9)	
PSP‐P	19 (27.1)	30 (36.6)	
PSP‐PI	9 (12.9)	10 (12.2)	
PSP‐PGF	0	1 (1.2)	
PSP‐SL	3 (4.3)	0	
PSP‐CBS	3 (4.3)	1 (1.2)	
PSP‐OM	1 (1.4)	1 (1.2)	
PSP‐F	7 (10.0)	5 (6.1)	
PSP‐C	1 (1.4)	2 (2.4)	
Unclassified	4 (5.7)	3 (3.7)	
Alternative diagnoses ruled out			0.44^b^
Parkinson’s disease	21 (30.0)	12 (14.6)	
Alzheimer’s disease	4 (5.7)	2 (2.4)	
Essential tremor	3 (4.3)	1 (1.2)	
Unspecified tremor	2 (2.9)	0	
Dementia with Lewy bodies	2 (2.9)	0	
Unspecified ataxia	1 (1.4)	1 (1.2)	
Multiple system atrophy	1 (1.4)	2 (2.4)	
Myelopathy	1 (1.4)	0	
Primary lateral sclerosis	1 (1.4)	0	
Sequelae of cervical myelopathy	1 (1.4)	0	
Normal‐pressure hydrocephalus	1 (1.4)	0	
Anterograde amnesia	1 (1.4)	0	
Transient ischemic attack	0	1 (1.2)	
Multifactorial dementia	0	1 (1.2)	
Corticobasal degeneration	0	1 (1.2)	
Wernicke’s disease	0	1 (1.2)	
Lacunar infarction in the basal ganglia	0	1 (1.2)	
VDRL testing performed			0.90^c^
Yes	53 (75.7)	58 (70.7)	
No	8 (11.4)	11 (13.4)	
CSF analysis performed			0.90^c^
Yes	12 (17.1)	11 (13.4)	
No	50 (71.4)	60 (73.2)	
Neuroimaging studies			
MRI	60 (85.7)	65 (79.3)	0.58^c^
Noncontrast CT	23 (32.9)	21 (25.6)	0.54^c^
Transcranial sonography (TCS)	11 (15.7)	6 (7.3)	0.23^c^
Contrast‐enhanced CT	3 (4.3)	1 (1.2)	0.41^b^
Magnetic resonance angiography (MRA)	1 (1.4)	1 (1.2)	0.80^b^
Neuroimaging findings			
Midbrain atrophy	26 (37.1)	46 (56.1)	0.23^b^
White matter microangiopathy	26 (37.1)	18 (22.0)	0.06^c^
Frontotemporal and/or cerebellar atrophy	15 (21.4)	12 (14.6)	0.36^c^
Lenticular hyperechogenicity and ventricular enlargement	1 (1.4)	2 (2.4)	0.40^b^
Type of therapy			0.09^c^
Monotherapy	13 (18.6)	8 (9.8)	
Polytherapy	57 (81.4)	71 (86.6)	
Medications used			
Levodopa/carbidopa	65 (92.9)	74 (90.2)	0.42^b^
SSRIs	50 (71.4)	60 (73.2)	0.10^b^
Amantadine	7 (10.0)	3 (3.7)	0.20^b^
Botulinum toxin	2 (2.9)	5 (6.1)	0.34^b^
Nonpharmacological management			0.003^b^
Psychotherapy	6 (8.6)	3 (3.7)	
Neurorehabilitation	30 (42.9)	22 (26.8)	
Psychotherapy plus neurorehabilitation	6 (8.6)	1 (1.2)	
Documented initial levodopa response			
Yes	22 (31.4)	12 (14.6)	—

*Note:* Data are presented as *n* (%) unless otherwise indicated. Percentages were calculated using available data for each variable; therefore, totals may not sum to 100% because of missing data. CSF, cerebrospinal fluid; PSP‐C, PSP with predominant cerebellar ataxia; PSP‐CBS, PSP with corticobasal syndrome; PSP‐F, PSP with predominant frontal presentation; PSP‐OM, PSP with predominant oculomotor dysfunction; PSP‐PI, PSP with predominant postural instability; PSP‐SL, PSP with predominant speech/language disorder; TCS, transcranial sonography.

Abbreviations: CT, computed tomography; MRA, magnetic resonance angiography; MRI, magnetic resonance imaging; PSP, progressive supranuclear palsy; PSP‐P, PSP‐parkinsonism; PSP‐PGF, PSP with progressive gait freezing; PSP‐RS, PSP‐Richardson syndrome; SSRIs, selective serotonin reuptake inhibitors; VDRL, Venereal Disease Research Laboratory.

^a^Mean ± standard deviation [median (interquartile range)].

^b^Fisher’s exact test.

^c^Pearson’s chi‐square test.

^d^Kruskal–Wallis test.

A family history of neurodegenerative disease was uncommon and showed no relevant differences by sex. PD was the most frequent initial alternative diagnosis documented in the cohort. Thirty‐three patients had initially received a diagnosis of PD and were later reclassified as PSP during clinical follow‐up. Of these, 14 patients were ultimately classified as PSP‐P (42.4%), 13 as PSP‐RS (39.4%), 4 as PSP‐PI (12.1%), 1 as PSP‐F (3.0%), and 1 as PSP‐SL (3.0%). Thus, most diagnostic revisions from PD to PSP occurred in patients ultimately classified as PSP‐P or PSP‐RS.

Among personal medical histories, arterial hypertension was the most common comorbidity, affecting 57.1% of women and 43.9% of men. Other cardiovascular comorbidities, including type 2 diabetes mellitus and dyslipidemia, showed similar frequencies across sexes. Hypothyroidism was significantly more frequent in women than in men (30.0% vs. 12.2%, *p* = 0.024), whereas psychoaffective disorders were comparably distributed. Sleep disorders were slightly more frequent in men, although without statistical significance.

Age at symptom onset was in the seventh decade of life in both sexes and did not differ significantly between women and men (71.5 ± 9.41 vs. 69.6 ± 8.32 years). By contrast, time from symptom onset to diagnosis was significantly longer in women than in men (3.98 ± 1.96 vs. 3.06 ± 2.32 years, *p* = 0.002).

PI was the most common presenting symptom in both sexes (35.7% in women and 36.6% in men). The second most frequent presenting symptom differed slightly by sex: akinesia or bradykinesia was more common in men (30.5% vs. 21.4%), whereas cognitive dysfunction was more frequent in women (28.6% vs. 15.9%). Oculomotor dysfunction was an uncommon presenting feature in both groups.

The most frequent clinical phenotype in women was PSP‐RS (32.9%), followed by PSP‐parkinsonism (PSP‐P) (27.1%). In men, PSP‐P was the most frequent phenotype (36.6%), followed by PSP‐RS (32.9%). PSP‐PI showed similar frequencies in both sexes, whereas other atypical phenotypes were uncommon. PSP‐SL was identified only in women, and PSP‐CBS was more frequent in women than in men. Frontally predominant cases were documented in both sexes, accounting for 10.0% of women and 6.1% of men. A small proportion of patients remained unclassified because of ambiguous clinical features or insufficient data. No significant sex‐related differences were observed in phenotype distribution.

No significant sex‐related differences were found in the frequency of VDRL testing or CSF analysis. Magnetic resonance imaging was the most frequently requested imaging study in both sexes, and midbrain atrophy was the most reported neuroimaging abnormality, followed by white matter microangiopathy and frontotemporal and/or cerebellar atrophy.

Therapeutic characteristics were largely similar between sexes. No significant differences were found in the type of therapy used or in the use of levodopa/carbidopa, selective serotonin reuptake inhibitors (SSRIs), amantadine, or botulinum toxin. Levodopa/carbidopa was prescribed to 139 of 152 patients (91.4%). Quantifiable dosing information was available for 100 of these treated patients; therefore, dose analyses were restricted to this subgroup. Daily doses were calculated using only the levodopa component. Among patients with available dosing data, men received a significantly higher initial levodopa dose than women (median [IQR], 300 [138] vs. 225 [100] mg/day; *p* = 0.007). However, no significant sex‐related differences were observed in the maximum documented daily levodopa dose (women vs. men: 550 [525] vs. 475 [450] mg/day; *p* = 0.15) or in the final recorded daily dose (475 [450] vs. 400 [450] mg/day;*p* = 0.23). Nonpharmacological management was significantly more frequent in women than in men, particularly neurorehabilitation and psychotherapy combined with neurorehabilitation. An initial therapeutic response to levodopa/carbidopa was documented in 34 treated patients, corresponding to 24.5% of those treated and 22.4% of the overall cohort.

Table 3 compares typical PSP (PSP‐RS), atypical PSP, and unclassified cases. Overall, most sociodemographic and clinical variables were similar across groups, with no significant differences in sex, age, age at symptom onset, comorbidities, presenting symptom, ancillary studies, treatment type, medication use, or levodopa response. However, two findings differed significantly across phenotypic groups. First, time from symptom onset to diagnosis was progressively longer in unclassified cases than in atypical PSP and typical PSP‐RS, suggesting greater diagnostic difficulty when the presentation does not clearly fit established phenotypes. Second, white matter microangiopathy and frontotemporal and/or cerebellar atrophy differed significantly among groups, whereas midbrain atrophy was frequent but similarly distributed. Although the comparison did not reach statistical significance, typical PSP‐RS more often presented with PI and akinesia/bradykinesia, whereas atypical PSP showed a higher proportion of cognitive dysfunction at onset. Detailed findings by individual PSP subtype are shown in the Supporting Information (Supporting Table S1).

**TABLE 3:** Sociodemographic and clinical characteristics according to PSP phenotype.

Variable	Typical PSP (PSP‐RS) (*n* = 50) *n* (%)	Atypical PSP (*n* = 95) *n* (%)	Unclassified (*n* = 7) *n* (%)	*p* value
Sex				0.83
Women	23 (46.0)	43 (45.3)	4 (57.1)	
Men	27 (54.0)	52 (54.7)	3 (42.9)	
Residence				0.46^b^
Metropolitan Area of Valle de Aburrá	44 (88.0)	75 (78.9)	4 (57.1)	
Outside the Metropolitan Area of Valle de Aburrá	5 (10.0)	18 (18.9)	3 (42.9)	
Age group, years				0.20^b^
< 55	1 (2.0)	0	0	
55–64	3 (6.0)	7 (7.4)	0	
65–74	13 (26.0)	25 (26.3)	2 (28.6)	
75–84	21 (42.0)	46 (48.4)	4 (57.1)	
85–94	10 (20.0)	14 (14.7)	1 (14.3)	
≥ 95	2 (4.0)	3 (3.2)	0	
Age, years^a^	77.5 ± 12.1 [79.5 (11.8)]	77.5 ± 8.6 [78.0 (12.0)]	77.3 ± 11.5 [83.0 (12.0)]	0.76
Age at symptom onset, years^a^	71.5 ± 8.3 [71.0 (11.75)]	70.1 ± 9.11 [68.0 (13.5)]	68.1 ± 9.8 [68.0 (11.0)]	0.57
Time from symptom onset to diagnosis, years^a^	2.9 ± 2.2 [2.0 (2.5)]	3.57 ± 2.0 [3.0 (3.0)]	7.3 ± 3.0 [8.0 (1.8)]	0.006
Personal medical history				
Hypertension	24 (48.0)	46 (48.4)	6 (85.7)	0.42
Type 2 diabetes mellitus	9 (18.0)	19 (20.0)	0	0.94^b^
Hypothyroidism	11 (22.0)	19 (20.0)	1 (14.3)	0.94^b^
Dyslipidemia	7 (14.0)	14 (14.7)	0	0.90^b^
> 3 cardiovascular comorbidities	7 (14.0)	12 (12.6)	2 (28.6)	0.78^b^
Psychoaffective disorders	12 (24.0)	8 (8.4)	0	0.09^b^
Sleep disorders	3 (6.0)	5 (5.3)	0	1.0^b^
Presenting symptom				0.08^b^
Postural instability	19 (38.0)	33 (34.7)	3 (42.9)	
Akinesia or bradykinesia	19 (38.0)	21 (22.1)	0	
Cognitive dysfunction	6 (12.0)	26 (27.4)	1 (14.3)	
Oculomotor dysfunction	2 (4.0)	4 (4.2)	0	
Other	4 (8.0)	9 (9.5)	3 (42.9)	
Alternative diagnoses ruled out				0.96^b^
Parkinson’s disease	13 (26.0)	20 (21.1)	0	
Alzheimer’s disease	1 (2.0)	5 (5.3)	0	
Essential tremor	1 (2.0)	2 (2.1)	0	
Unspecified tremor	0	2 (2.1)	0	
Dementia with Lewy bodies	1 (2.0)	1 (1.1)	0	
Unspecified ataxia	0	2 (2.1)	0	
Multiple system atrophy	0	0	0	
Myelopathy	0	1 (1.1)	0	
Primary lateral sclerosis	0	0	1 (14.3)	
Sequelae of cervical myelopathy	1 (2.0)	0	0	
Normal‐pressure hydrocephalus	0	1 (1.1)	0	
Anterograde amnesia	0	1 (1.1)	0	
Transient ischemic attack	0	0	1 (14.3)	
Multifactorial dementia	0	1 (1.1)	0	
Corticobasal degeneration	0	1 (1.1)	0	
Wernicke’s disease	0	1 (1.1)	0	
Lacunar infarction in the basal ganglia	1 (2.0)	1 (1.1)	0	
VDRL testing performed	38 (76.0)	68 (71.6)	5 (71.4)	0.55^b^
CSF analysis performed	11 (22.0)	17 (17.9)	0	0.56^b^
Neuroimaging studies				
MRI	42 (84.0)	76 (80.0)	7 (100)	0.89^b^
Noncontrast CT	18 (36.0)	23 (24.2)	4 (57.1)	0.48^b^
Transcranial sonography (TCS)	7 (14.0)	8 (8.4)	2 (28.6)	0.35^b^
Contrast‐enhanced CT	2 (4.0)	2 (2.1)	0	0.71^b^
Magnetic resonance angiography (MRA)	2 (4.0)	0	0	0.29^b^
Neuroimaging findings				
Lenticular hyperechogenicity and ventricular enlargement	1 (2.0)	2 (2.1)	0	0.50^b^
White matter microangiopathy	14 (28.0)	27 (28.4)	3 (42.9)	0.017^b^
Frontotemporal and/or cerebellar atrophy	12 (24.0)	13 (13.7)	2 (28.6)	0.006^b^
Midbrain atrophy	23 (46.0)	41 (43.2)	2 (28.6)	0.51^b^
Type of therapy				0.05^b^
Monotherapy	3 (6)	17 (17.9)	1 (14.3)	
Polytherapy	46 (92)	77 (81.1)	5 (61.4)	
Medications used				
Levodopa/carbidopa	48 (96.0)	86 (90.5)	5 (71.4)	0.09^b^
SSRIs	42 (84.0)	70 (73.7)	4 (57.1)	0.23^b^
Amantadine	2 (4.0)	6 (6.3)	2 (28.6)	0.20^b^
Botulinum toxin	1 (2.0)	6 (6.3)	0	0.67^b^
Nonpharmacological management				0.87^b^
Psychotherapy	3 (6.0)	6 (6.3)	0	
Neurorehabilitation	19 (38.0)	29 (30.5)	4 (57.1)	
Psychotherapy plus neurorehabilitation	3 (6.0)	4 (4.2)	0	
Documented initial levodopa response				
Yes	11 (22.0)	22 (23.2)	1 (14.3)	

*Note:* Data are presented as *n* (%) unless otherwise indicated. Percentages were calculated using available data for each variable; therefore, totals may not sum to 100% because of missing data. CSF, cerebrospinal fluid; TCS, transcranial sonography.

Abbreviations: CT, computed tomography; MRA, magnetic resonance angiography; MRI, magnetic resonance imaging; PSP, progressive supranuclear palsy; PSP‐RS, progressive supranuclear palsy‐Richardson syndrome; SSRIs, selective serotonin reuptake inhibitors; VDRL, Venereal Disease Research Laboratory.

^a^Mean ± standard deviation [median (interquartile range)].

^b^Fisher’s exact test.

## 4. Discussion

This retrospective, observational, cross‐sectional study was based on a census of all patients with a clinical diagnosis of PSP evaluated at a neurological referral center in Antioquia between 2013 and 2023. In a national setting where published evidence on PSP remains limited to isolated case reports and very small series, our study provides a systematic clinical and sociodemographic characterization of a relatively large cohort derived from detailed medical record review. In addition to estimating the period prevalence of PSP in the adult population of the department, we examined differences according to sex and clinical presentation (typical vs. atypical PSP), as well as potential associations between sociodemographic and clinical variables. Taken together, these findings expand the limited regional evidence on PSP and allow comparison with data reported in the international literature.

Our overall prevalence of 1.8 per 100,000 inhabitants (2.04 per 100,000 in men and 1.52 per 100,000 in women) lies at the lower end of the international estimates reported to date. Kawashima et al. [19] reported a population prevalence of 5.82 per 100,000 in Japan. In the United Kingdom, Swallow et al. [8] in a systematic review of prevalence studies identified a pooled PSP prevalence of 7.1 per 100,000 among the highest‐quality studies. Likewise, Lyons et al. [20] reported a pooled prevalence estimate of 6.92 per 100,000 and noted that published PSP prevalence estimates have ranged from 1.0 to 18 per 100,000 across studies. More recently, Kukkle et al. [21] summarized global prevalence estimates in a narrower range of approximately 5 to 6.4 per 100,000. Taking together, our findings are broadly consistent with international literature, although they may suggest underascertainment or methodological differences in case identification in our setting.

In the present series, 53.9% of patients with PSP were men, indicating a slight male predominance. This finding is consistent with some population‐based epidemiological studies that have reported either a higher proportion of men or an approximately balanced sex distribution. In prevalence studies from the United Kingdom and Scotland, the proportion of men was approximately 52%–55% [22, 23], whereas population‐based incidence analyses from the United States also found higher rates in men than in women [24]. However, the evidence is not uniform, and other series have shown no male predominance or have even reported a higher proportion of women, as described in a study from Israel in which 53% of cases were female [25] and in the report by Baba et al. [26]. This heterogeneity has also been supported by recent systematic reviews and meta‐analyses, which suggest that there is no consistent or universal sex‐related difference in PSP [20]. Taken together, the sex distribution observed in our cohort is in keeping with the variability described internationally and suggests that any sex predominance in PSP is, at most, modest. Nevertheless, in addition to methodological and case‐ascertainment factors, plausible biological explanations should also be considered. In particular, findings from the ENGENE‐PSP study have raised the possibility that estrogen exposure may exert a protective effect on PSP susceptibility and/or severity through mechanisms related to tau pathology and neuroinflammation [27]. However, this hypothesis does not fully account for the phenotypic differences observed between sexes, and prospective studies with systematic assessment of hormonal history, biomarkers, and longitudinal clinical characterization are still needed [7].

With regard to age, the greatest disease burden in our cohort was concentrated in the 75‐ to 84‐year age group, and the mean age at evaluation was 80.1 years in women and 75.2 years in men. These findings are broadly consistent with the literature, which places PSP onset and clinical presentation predominantly in the seventh and eighth decades of life. Systematic and narrative reviews have reported mean ages at onset generally ranging from the early 60s to early 70s, with higher prevalence and case concentration in older age groups, particularly after 70 years of age [8, 19, 20]. Consistently, the global review by Kukkle et al. [21] summarizes a typical age range for presentation between 63 and 77 years, with a higher prevalence in those over 70. Overall, our data align with this epidemiological pattern, showing a predominance in older age groups and a concentration in the eighth decade.

The age‐ and sex‐specific prevalence findings reinforce the need for closer clinical surveillance in older adults, particularly in the 75‐ to 84‐year age group, where disease burden was highest. With regard to age at symptom onset, women in our cohort showed a later onset than men (71 vs. 67 years), with overall onset occurring in the seventh decade of life. This range is consistent with that reported in international series. For example, Arena et al. [28] reported a median age at onset of 65.9 years (IQR, 60.6–70.0), and global reviews have generally described a mean age at onset ranging from 63 to 72 years [21]. The slightly later age at onset observed in women in our cohort may reflect local demographic differences, variations in access to health care, or survival bias, rather than a true biological modification of the course of the tauopathy.

With regard to the interval between symptom onset and diagnosis, patients in our cohort showed a mean delay of 3.5 years, a figure that is consistent with the diagnostic delay reported in the literature, which is generally around 3–4 years [25, 29]. This interval has been attributed to the insidious nature of the initial clinical presentation, the phenotypic overlap with PD in the early stages, particularly in PSP‐P, and the progressive emergence of cardinal features such as vertical supranuclear gaze palsy and early falls [8, 20, 21].

The finding that 33 patients were initially diagnosed with PD and later reclassified as PSP further supports the diagnostic overlap between early PSP and PD, particularly in PSP‐P and PSP‐RS phenotypes. This observation reinforces the importance of longitudinal follow‐up and reassessment of patients with atypical parkinsonism, especially when PI, supranuclear gaze abnormalities, early cognitive or frontal symptoms, or poor levodopa response emerge over time.

When examining the initial clinical presentation, we found that PI was the most frequent presenting symptom in both women and men, a finding consistent with the classic description of PSP‐RS, in which early falls and axial instability are among the predominant initial manifestations [9, 30]. Bradykinesia was more common as the inaugural symptom in men than in women, a pattern consistent with series describing a substantial proportion of patients with a PSP‐P presentation, characterized by early parkinsonism and frequent clinical overlap with PD in the initial stages [28, 31]. Notably, cognitive dysfunction as the presenting symptom was more frequent in women than in men, which is in line with the growing evidence that frontal and behavioral manifestations may precede the classic motor signs in a significant subgroup of patients, within the spectrum of PSP‐F or other variants with early frontosubcortical involvement [9, 30]. In contrast, oculomotor symptoms were uncommon as the initial manifestation (< 5%), which is consistent with clinical reviews showing that vertical supranuclear gaze palsy usually emerges progressively and does not necessarily constitute the first symptom recognized by the patient [31]. Taken together, these findings reflect a heterogeneous distribution of presenting symptoms, in keeping with the phenotypic variability of PSP described in the contemporary literature, and underscore the importance of considering nonclassical presentations in the early stages in order to reduce diagnostic delay.

Building on this heterogeneity of presenting symptoms, the overall phenotypic distribution in our cohort, integrating women and men, showed a distinctive pattern: PSP‐RS and PSP‐P occurred at similar frequencies, followed by PSP‐PI and PSP‐F, whereas the remaining variants were uncommon and 4.6% of cases remained unclassifiable. This finding suggests a relatively lower representation of PSP‐RS than in several international series, in which PSP‐RS usually accounts for the majority of cases when MDS criteria are applied. For example, Mahale et al. [32] reported frequencies of 71.7% for PSP‐RS and 13.4% for PSP‐P, with lower proportions of PSP‐CBS, PSP‐F, PSP‐PGF, PSP‐PI, and PSP‐SL. Similarly, in the multinational study by Pillas et al. [33] (*n* = 892), application of the MAX rules and incorporation of signs observed throughout the full clinical course increased the proportion of PSP‐RS to 75%, whereas PSP‐P and PSP‐PI accounted for only 3% and 2%, respectively. In contrast, cohorts that phenotype patients at earlier stages tend to show greater diversity. In Tunisia, Nasri et al. [34] (*n* = 112) reported PSP‐RS in 42.85% of cases, with relevant proportions of cortical and subcortical phenotypes, including PSP‐CBS (17.6%), PSP‐F (9.4%), PSP‐SL (8.2%), PSP‐P (11.6%), and PSP‐PI (8%). This pattern is consistent with the notion that PSP subtypes may evolve over time and that many patients ultimately fulfill criteria for PSP‐RS.

In this cohort, approximately 11% of patients with PSP had a documented family history of neurodegenerative disease, mainly Alzheimer’s disease, dementia, and PD. This frequency is consistent with that reported in international series with neuropathological confirmation, in which approximately 15% of patients with PSP have a family history of parkinsonism or dementia [35], and is lower than that described in studies that performed a systematic assessment of family history among first‐degree relatives, where frequencies reached up to 33% [36]. These differences are likely attributable, to a large extent, to methodological variation in the definition and ascertainment of family history.

The retrospective design of this study, based on medical record review, limits the accuracy of family history ascertainment, as this information was not consistently documented across records, suggesting probable underreporting. Nonetheless, the identification of a positive family history in nearly one in eight patients supports the view that, although PSP is predominantly a sporadic disorder, shared genetic susceptibility may contribute to the familial aggregation of different neurodegenerative phenotypes, in keeping with the genetic architecture described for PSP [37, 38]. This finding further underscores the importance of systematically recording family history during the clinical assessment of patients with PSP.

In our study, vascular and metabolic comorbidities were common but did not differ significantly by sex, with the exception of hypothyroidism. Arterial hypertension was the most prevalent condition, followed by diabetes mellitus and dyslipidemia. The burden of ≥ 3 cardiovascular comorbidities was comparable between groups (14.3% vs. 13.4%). These findings are consistent with previous clinicopathological series suggesting that vascular risk factors are common in patients with PSP but are unlikely to play a primary etiopathogenic role [39, 40]. However, Rabadia et al. [39] reported a modest but statistically significant association between hypertension and PSP (odds ratio, 1.492; 95% confidence interval, 1.045–2.129; *p* = 0.027). Notably, hypothyroidism was significantly more frequent in women, a difference that likely reflects the well‐known sex distribution of thyroid disease in the general population rather than a PSP‐specific association. To our knowledge, only Zella et al. [41] have reported thyroid disorders in a PSP cohort, identifying them in 57 of 350 patients (17.0%).

Large neuropathological studies have emphasized that PSP is a primary 4‐repeat tauopathy, with limited evidence supporting a causal contribution of systemic vascular comorbidities [41, 42]. Therefore, although cardiovascular and metabolic conditions were common in our cohort, their distribution likely reflects background population risk and supports the view that comorbidities in PSP are more likely age‐related epiphenomena than true disease‐modifying factors.

About neuroimaging findings, midbrain atrophy was the most frequent abnormality identified on MRI, and frontotemporal and/or cerebellar atrophy was also reported. This pattern is consistent with literature. Bacchi et al. [43] showed that several MRI markers, particularly quantitative midbrain‐based measures such as midbrain area and the Magnetic Resonance Parkinsonism Index (MRPI), can achieve high diagnostic sensitivity and specificity for PSP, supporting midbrain atrophy as the most robust structural marker on conventional MRI. In keeping with this, recent reviews have emphasized that midbrain atrophy remains the cardinal neuroimaging feature of PSP and a central supportive finding in the diagnostic workup [44]. At the same time, these studies also recognize that neurodegeneration in PSP is not confined to the brainstem, but may extend to frontal regions and cerebellar structures, in line with the involvement of frontosubcortical and dentato‐rubro‐thalamic circuits. Accordingly, the presence of frontotemporal and cerebellar atrophy in our series may reflect the anatomical spread of tau pathology beyond the midbrain and may contribute to the clinical heterogeneity observed across PSP phenotypes.

A substantial proportion of patients showed white matter microangiopathy on neuroimaging. Current evidence suggests that cerebral small vessel disease often coexists with PSP but generally represents an age‐related comorbid process rather than a primary cause of PSP [45]. Although it may have limited prognostic relevance, particularly when temporal lobe white matter changes are present, it does not displace tau pathology as the central disease mechanism [46].

Only a small number of patients in our cohort underwent TCS. According to Wen et al. [47], TCS may serve as a complementary tool in the evaluation of parkinsonian disorders, particularly through the detection of lenticular nucleus hyperechogenicity and ventricular enlargement. Their meta‐analysis showed that lenticular nucleus hyperechogenicity is a relatively frequent finding in PSP and more prevalent than substantia nigra hyperechogenicity, suggesting a sonographic pattern more closely associated with atypical parkinsonism than with idiopathic PD. Ventricular enlargement may likewise reflect structural changes related to the diencephalic and mesencephalic atrophy that characterizes the disease. However, TCS should be regarded as an adjunctive rather than a primary diagnostic technique, as its diagnostic performance remains inferior to that of structural MRI markers.

In our series, nontreponemal serologic testing (VDRL) was frequently requested during the initial diagnostic workup, whereas CSF analysis was substantially less common; moreover, no statistically significant differences were observed according to sex or clinical PSP phenotype. These findings are consistent with the available literature, in which the diagnosis of PSP remains based primarily on clinical and radiological criteria, whereas fluid studies are not part of the routine confirmation of the disease but are mainly reserved for excluding alternative diagnoses when atypical features are present in the clinical history, neurological examination, or neuroimaging [11, 48]. In this context, the relatively frequent use of VDRL testing in our cohort may be interpreted as a strategy to exclude potentially treatable causes of PSP‐like syndromes, given that neurosyphilis, although uncommon, has been described as a PSP mimic in case reports and reviews of the differential diagnosis [49, 50]. By contrast, the lower frequency of lumbar puncture suggests a selective use of CSF analysis, likely restricted to patients with diagnostic uncertainty, atypical progression, or suspicion of infectious, inflammatory, or prion‐related etiologies, rather than as part of a universal initial evaluation protocol [11, 48].

Another relevant differential diagnosis is anti‐IgLON5 disease, an increasingly recognized autoimmune‐neurodegenerative disorder that may present with PSP‐like features, including supranuclear gaze palsy, gait instability, parkinsonism, bulbar symptoms, cognitive impairment, and prominent sleep disturbances. This diagnosis should be considered particularly when PSP‐like manifestations coexist with parasomnia, sleep‐disordered breathing, dysautonomia, stridor, rapid or atypical progression, or other clinical red flags suggestive of an immune‐mediated process [51, 52]. In the present retrospective cohort, autoimmune antibody testing, including anti‐IgLON5 antibodies, was not systematically performed. This limitation should be interpreted in the context of the 2013–2023 study period, during which anti‐IgLON5 disease was still an emerging diagnostic entity, and in the context of limited routine access to specialized autoimmune neurology testing in our setting. Therefore, although alternative diagnoses were evaluated according to clinical suspicion, anti‐IgLON5 disease and other immune‐mediated PSP mimics could not be formally excluded in all cases.

In our study, the therapeutic management of PSP was predominantly symptomatic and consistent with real‐world clinical practice. Levodopa/carbidopa was the most frequently used medication, followed by SSRIs, whereas amantadine and botulinum toxin were prescribed far less frequently. This pattern reinforces that, in the absence of disease‐modifying therapies, levodopa remains the main pharmacological option for motor symptoms in PSP, particularly bradykinesia and rigidity, despite its limited and often transient benefit [14, 42, 53–56]. The use of levodopa/carbidopa in our cohort was higher than that reported in large multinational cohorts, where levodopa has been prescribed in 54.4%–67.7% of patients [42, 53]. This may reflect real‐world prescribing practices in a referral setting, frequent diagnostic overlap with PD, and the limited availability of alternative symptomatic therapies. Nevertheless, levodopa responsiveness should be interpreted cautiously in retrospective cohorts. Previous studies have shown that only a subset of patients with PSP experience clinical benefit, usually modest (20%–40%); moreover, when levodopa response has been assessed objectively using an acute levodopa challenge and the MDS‐UPDRS part III, only 4.2% of patients achieve a clinically meaningful response [57, 58]. Expert recommendations further suggest that nonresponsiveness should not be concluded unless an adequate trial has been performed, usually with doses in the range of 750–1000 mg/day or up to 1 g/day when tolerated [14, 54]. Therefore, limited or absent documented benefit in our cohort should not be interpreted as definitive levodopa refractoriness, particularly because dose escalation, adherence, treatment duration, and response assessment could not be standardized retrospectively.

Likewise, the high use of SSRIs in our series exceeded the 35.1% reported by Iyer et al. [53], probably reflecting a substantial burden of affective and behavioral symptoms. Nonpharmacological management differed significantly by sex, with women more frequently receiving neurorehabilitation and combined psychotherapy plus neurorehabilitation. This finding is relevant because neurorehabilitation remains underused in PSP, despite CurePSP consensus recommendations that it should begin at diagnosis and evidence that fewer than half of patients gain access to it [57, 59]. Overall, our findings support that PSP treatment remains largely empirical, symptomatic, and of modest effectiveness while highlighting gaps in access to rehabilitation‐based interventions.

Comparison of typical, atypical, and unclassified PSP showed that most sociodemographic and clinical variables were broadly similar across subgroups, in keeping with previous reports [60–62]. The most relevant finding was the longer disease duration before diagnosis in unclassified cases, suggesting that the absence of a recognizable phenotype constitutes a major diagnostic barrier. This observation is consistent with the literature documenting prolonged diagnostic delays, a high frequency of alternative initial diagnoses, and a substantial proportion of patients who remain unclassifiable during the early stages of the disease [29, 63, 64]. From a neuroimaging perspective, the comparable frequency of midbrain atrophy across groups, although lower in unclassified cases, supports the notion that this marker may be less prominent outside PSP‐RS or in earlier disease stages [65]. By contrast, the higher proportion of white matter microangiopathy in unclassified cases raises the possibility that small vessel disease may contribute to phenotypic complexity and diagnostic delay, a plausible interpretation given that greater white matter hyperintensity burden has been associated with more severe motor impairment and functional disability in PSP [66]. In addition, the trend toward more frequent cognitive onset in atypical PSP, although not statistically significant, is consistent with variants such as PSP‐F and PSP‐CBS, in which frontal or corticobasal features may precede the classic manifestations of the disease by several years [15, 61].

This study has several limitations that should be considered when interpreting its findings. First, its retrospective design relied on historical medical records, the quality and completeness of which varied over time, potentially affecting phenotypic classification. The inherent clinical heterogeneity of PSP complicates diagnostic categorization during life. Williams et al. [67] showed in 103 neuropathologically confirmed cases that 14% could not be assigned to any defined phenotype and that the classic description of the disease did not adequately capture approximately one‐third of cases. Similarly, Respondek et al. [68] reported in a multicenter retrospective cohort of 100 postmortem‐confirmed cases that more than half displayed overlapping features or did not fit proposed classification schemes. Although the MDS‐PSP criteria have broadened the recognized phenotypic spectrum, as documented by Höglinger et al. [11] and Boxer et al. [69], their application in clinical cohorts without neuropathological confirmation inevitably introduces diagnostic uncertainty. Second, the absence of histopathological confirmation in most cases limits diagnostic certainty, particularly because clinically atypical phenotypes may be underrepresented or misclassified in referral‐based cohorts.

From a statistical standpoint, collinearity was identified among several independent variables, and principal component analysis (PCA) was explored as a dimensionality‐reduction strategy in accordance with prior methodological recommendations [17, 70]. However, generation of component scores required exclusion of cases with missing data in the original variables, resulting in loss of valid observations in one category of the dependent variable and preventing model convergence. Consequently, we were unable to estimate a multivariable model capable of quantifying the independent contribution of each predictor or adequately controlling for confounding among correlated variables. The comparative analyses presented here are, therefore, bivariate and should be interpreted within that limitation. In addition, sample size imposed constraints on the number of predictors that could be examined simultaneously, in keeping with events‐per‐variable considerations described by Vittinghoff and McCulloch [71].

Future studies should adopt prospective longitudinal designs that allow systematic and standardized collection of clinical and sociodemographic data from the time of diagnosis onward, thereby overcoming the constraints inherent to retrospective data capture. Larger sample sizes will be necessary to provide sufficient statistical power for stable multivariable modeling and to identify independent predictors of typical versus atypical clinical presentation while appropriately accounting for confounding among correlated variables. Whenever feasible, incorporation of neuropathological confirmation through brain donation programs would strengthen diagnostic certainty and allow direct evaluation of the validity of clinical criteria applied during life, particularly given the marked phenotypic heterogeneity of PSP. Multicenter studies across Latin America would also improve the representativeness of findings and facilitate comparison of clinical and demographic characteristics across different health care settings in the region. Finally, because our results confirm that PSP continues to be diagnosed several years after symptom onset, consistent with international series, future research should focus on the development and validation of early screening tools and clinically based diagnostic algorithms aimed at reducing diagnostic latency, enabling earlier symptomatic intervention, and improving anticipatory care planning in less advanced stages of disease.

## 5. Conclusion

This study provides a clinical and epidemiological characterization of PSP in a Colombian referral center cohort and contributes to the limited data available from Latin America. In our setting, PSP predominantly affected older adults and was characterized by phenotypic heterogeneity, delayed diagnosis, and largely symptomatic management. The longer diagnostic latency observed in unclassified cases highlights the challenges posed by nonclassical presentations in routine clinical practice. These findings underscore the need for earlier recognition, more structured diagnostic pathways, and greater integration of multidisciplinary and rehabilitation‐based care for patients with PSP.

## Funding

This study was supported by Universidad Cooperativa de Colombia, INV3727.

## Conflicts of Interest

The authors declare no conflicts of interest.

## Supporting Information

Additional supporting information can be found online in the Supporting Information section.

## Supporting information


**Supporting Information** Supporting Table S1. Sociodemographic and clinical characteristics according to progressive supranuclear palsy phenotype. This table presents additional descriptive data on demographic variables, clinical presentation, diagnostic work‐up, neuroimaging findings, treatment, and levodopa response across PSP phenotypes. Percentages were calculated using available data for each variable and may not sum to 100% because of missing data.

## Data Availability

The data supporting the findings of this study are available from the corresponding author upon reasonable request, subject to applicable ethical, institutional, and patient confidentiality requirements.

## References

[1] Steele J. C. , Richardson J. C. , and Olszewski J. , Progressive Supranuclear Palsy. A Heterogeneous Degeneration Involving the Brain Stem, Basal Ganglia and Cerebellum With Vertical Gaze and Pseudobulbar Palsy, Nuchal Dystonia and Dementia, Archives of Neurology. (1964) 10, 333–359, 10.1001/archneur.1964.00460160003001. 14107684

[2] Kovacs G. G. , Lukic M. J. , Irwin D. J. et al., Distribution Patterns of Tau Pathology in Progressive Supranuclear Palsy, Acta Neuropathologica. (2020) 140, no. 2, 99–119, 10.1007/s00401-020-02158-2. PMC7360645 32383020

[3] Factor S. A. and Esper C. D. , Progressive Supranuclear Palsy (PSP): Clinical Features and Diagnosis, 2020, Wolters Kluwer, Waltham, MA.

[4] Golbe L. I. , Davis P. H. , Schoenberg B. S. , and Duvoisin R. C. , Prevalence and Natural History of Progressive Supranuclear Palsy, Neurology. (1988) 38, no. 7, 1031–1034, 10.1212/wnl.38.7.1031. 3386818

[5] Schrag A. , Ben-Shlomo Y. , and Quinn N. P. , Prevalence of Progressive Supranuclear Palsy and Multiple System Atrophy: A Cross-Sectional Study, Lancet (London, England). (1999) 354, no. 9192, 1771–1775, 10.1016/s0140-6736(99)04137-9. 10577638

[6] Erkkinen M. G. , Kim M. O. , and Geschwind M. D. , Clinical Neurology and Epidemiology of the Major Neurodegenerative Diseases, Cold Spring Harbor Perspectives in Biology. (2018) 10, no. 4, 10.1101/cshperspect.a033118. PMC5880171 28716886

[7] Mahale R. R. , Krishnan S. , Divya K. P. , Jisha V. T. , and Kishore A. , Gender Differences in Progressive Supranuclear Palsy, Acta Neurologica Belgica. (2022) 122, no. 2, 357–362, 10.1007/s13760-021-01599-0. 33595832

[8] Swallow D. M. A. , Zheng C. S. , and Counsell C. E. , Systematic Review of Prevalence Studies of Progressive Supranuclear Palsy and Corticobasal Syndrome, Movement Disorders Clinical Practice. (2022) 9, no. 5, 604–613, 10.1002/mdc3.13489. PMC9274340 35844273

[9] Parthimos T. P. and Schulpis K. H. , The Progressive Supranuclear Palsy: Past and Present Aspects, Clinical Gerontologist. (2020) 43, no. 2, 155–180, 10.1080/07317115.2019.1694115. 31752626

[10] Park H. K. , Ilango S. D. , and Litvan I. , Environmental Risk Factors for Progressive Supranuclear Palsy, Journal of movement disorders. (2021) 14, no. 2, 103–113, 10.14802/jmd.20173. PMC8175813 34062646

[11] Höglinger G. U. , Respondek G. , Stamelou M. et al., Clinical Diagnosis of Progressive Supranuclear Palsy: The Movement Disorder Society Criteria, Movement Disorders. (2017) 32, no. 6, 853–864, 10.1002/mds.26987. PMC5516529 28467028

[12] Ichikawa-Escamilla E. , Velasco-Martínez R. A. , and Adalid-Peralta L. , Progressive Supranuclear Palsy Syndrome: An Overview, IBRO neuroscience reports. (2024) 16, 598–608, 10.1016/j.ibneur.2024.04.008. PMC11126858 38800085

[13] Gómez-Virgilio L. , Gutiérrez-Malacara A. I. , Rivera-Osorio J. et al., Progressive Supranuclear Palsy: An Updated Approach on Diagnosis, Treatment, Risk Factors and Outlook in Mexico, Gaceta Médica de México. (2024) 160, no. 4, 374–383, 10.24875/gmm.m24000930. 39832321

[14] Coughlin D. G. and Litvan I. , Progressive Supranuclear Palsy: Advances in Diagnosis and Management, Parkinsonism & Related Disorders. (2020) 73, 105–116, 10.1016/j.parkreldis.2020.04.014. PMC7462164 32487421

[15] Lopez G. , Bayulkem K. , and Hallett M. , Progressive Supranuclear Palsy (PSP): Richardson Syndrome and Other PSP Variants, Acta Neurologica Scandinavica. (2016) 134, no. 4, 242–249, 10.1111/ane.12546. PMC7292631 27070344

[16] DANE , Geovisor Registro Estadístico Base De Población-REBP, 2018.

[17] Aguilera A. M. , Escabias M. , and Valderrama M. J. , Using Principal Components for Estimating Logistic Regression With High-Dimensional Multicollinear Data, Computational Statistics & Data Analysis. (2006) 50, no. 8, 1905–1924, 10.1016/j.csda.2005.03.011.

[18] Zhang Z. and Castelló A. , Principal Components Analysis in Clinical Studies, Annals of Translational Medicine. (2017) 5, no. 17, 10.21037/atm.2017.07.12. PMC5599285 28936445

[19] Kawashima M. , Miyake M. , Kusumi M. , Adachi Y. , and Nakashima K. , Prevalence of Progressive Supranuclear Palsy in Yonago, Japan, Movement Disorders. (2004) 19, no. 10, 1239–1240, 10.1002/mds.20149. 15390010

[20] Lyons S. , Trépel D. , Lynch T. , Walsh R. , and O’Dowd S. , The Prevalence and Incidence of Progressive Supranuclear Palsy and Corticobasal Syndrome: A Systematic Review and Meta-Analysis, Journal of Neurology. (2023) 270, no. 9, 4451–4465, 10.1007/s00415-023-11791-2. PMC10421779 37289323

[21] Kukkle P. L. , Neupane R. , Pantelyat A. et al., Progressive Supranuclear Palsy—A Global Review, Movement Disorders Clinical Practice. (2025) 13, no. 3, 611–622, 10.1002/mdc3.70338. PMC13042570 40898879

[22] Nath U. , Ben-Shlomo Y. , Thomson R. G. et al., The Prevalence of Progressive Supranuclear Palsy (Steele-Richardson-Olszewski Syndrome) in the UK, Brain: A Journal of Neurology. (2001) 124, no. Pt 7, 1438–1449, 10.1093/brain/124.7.1438. 11408338

[23] Swallow D. M. A. and Counsell C. E. , Prevalence of Progressive Supranuclear Palsy and Corticobasal Syndrome in Scotland, Neuroepidemiology. (2022) 56, no. 4, 291–297, 10.1159/000525334. 35654007

[24] Stang C. D. , Turcano P. , Mielke M. M. et al., Incidence and Trends of Progressive Supranuclear Palsy and Corticobasal Syndrome: a Population-based Study, Journal of Parkinson’s Disease. (2020) 10, no. 1, 179–184, 10.3233/jpd-191744. PMC7153436 31594251

[25] Barer Y. , Chodick G. , Cohen R. et al., Epidemiology of Progressive Supranuclear Palsy: Real World Data from the Second Largest Health Plan in Israel, Brain Sciences. (2022) 12, no. 9, 10.3390/brainsci12091126. PMC9496895 36138862

[26] Baba Y. , Putzke J. D. , Whaley N. R. , Wszolek Z. K. , and Uitti R. J. , Progressive Supranuclear Palsy: Phenotypic Sex Differences in a Clinical Cohort, Movement Disorders: Official Journal of the Movement Disorder Society. (2006) 21, no. 5, 689–692, 10.1002/mds.20769. 16342256

[27] Park H. K. , Ilango S. , Charriez C. M. et al., Lifetime Exposure to Estrogen and Progressive Supranuclear Palsy: Environmental and Genetic PSP Study, Movement Disorders. (2018) 33, no. 3, 468–472, 10.1002/mds.27336. PMC5840026 29460982

[28] Arena J. E. , Weigand S. D. , Whitwell J. L. et al., Progressive Supranuclear Palsy: Progression and Survival, Journal of Neurology. (2016) 263, no. 2, 380–389, 10.1007/s00415-015-7990-2. 26705121

[29] Mamarabadi M. , Razjouyan H. , and Golbe L. I. , Is the Latency From Progressive Supranuclear Palsy Onset to Diagnosis Improving?, Movement Disorders Clinical Practice. (2018) 5, no. 6, 603–606, 10.1002/mdc3.12678. PMC6277372 30637280

[30] Morgan J. C. , Ye X. , Mellor J. A. et al., Disease Course and Treatment Patterns in Progressive Supranuclear Palsy: A Real-World Study, Journal of the Neurological Sciences. (2021) 421, 10.1016/j.jns.2020.117293. 33385754

[31] Fimbres-Laborin F. M. , Carrillo-Mezo R. , Lopez de la Cruz R. , and Boll M. C. , Progressive Supranuclear Palsy: Clinical Features and Neuroimaging in a Case Series, Cureus. (2025) 17, no. 5, 10.7759/cureus.83588. PMC12140374 40476102

[32] Mahale R. R. , Krishnan S. , Divya K. P. , Jisha V. T. , and Kishore A. , Subtypes of PSP and Prognosis: A Retrospective Analysis, Annals of Indian Academy of Neurology. (2021) 24, no. 1, 56–62, 10.4103/aian.AIAN_611_20. PMC8061531 33911380

[33] Pillas D. , Klein A. , Gasalla T. et al., Phenotypic Variants of Progressive Supranuclear Palsy in a Real-World Dataset as Identified by Existing Algorithms, Journal of the Neurological Sciences. (2025) 473, 10.1016/j.jns.2025.123519. 40339436

[34] Nasri A. , Sghaier I. , Neji A. et al., Phenotypic Spectrum of Progressive Supranuclear Palsy: Clinical Study and Apolipoprotein E Effect, Journal of movement disorders.(2024) 17, no. 2, 158–170, 10.14802/jmd.23178. PMC11082606 38290492

[35] Fujioka S. , Algom A. A. , Murray M. E. et al., Similarities Between Familial and Sporadic Autopsy-Proven Progressive Supranuclear Palsy, Neurology. (2013) 80, no. 22, 2076–2078, 10.1212/WNL.0b013e318294b2eb. PMC3716405 23635960

[36] Kaat L. D. , Boon A. J. W. , Azmani A. et al., Familial Aggregation of Parkinsonism in Progressive Supranuclear Palsy, Neurology. (2009) 73, no. 2, 98–105, 10.1212/WNL.0b013e3181a92bcc. 19458322

[37] Höglinger G. U. , Melhem N. M. , Dickson D. W. et al., Identification of Common Variants Influencing Risk of the Tauopathy Progressive Supranuclear Palsy, Nature Genetics. (2011) 43, no. 7, 699–705, 10.1038/ng.859. PMC3125476 21685912

[38] Im S. Y. , Kim Y. E. , and Kim Y. J. , Genetics of Progressive Supranuclear Palsy, Journal of Movement Disorders. (2015) 8, no. 3, 122–129, 10.14802/jmd.15033. PMC4572662 26413239

[39] Rabadia S. V. , Litvan I. , Juncos J. et al., Hypertension and Progressive Supranuclear Palsy, Parkinsonism & Related Disorders. (2019) 66, 166–170, 10.1016/j.parkreldis.2019.07.036. 31420308

[40] Jellinger K. A. , Comorbid Pathologies and Their Impact on Progressive Supranuclear Palsy: Current View, Journal of Neural Transmission. (2025) 133, no. 5, 831–841, 10.1007/s00702-025-03005-y. 40794238

[41] Zella M. A. S. , Bartig D. , Herrmann L. et al., Hospitalization Rates and Comorbidities in Patients With Progressive Supranuclear Palsy in Germany From 2010 to 2017, Journal of Clinical Medicine. (2020) 9, no. 8, 10.3390/jcm9082454. PMC7465231 32751888

[42] Greten S. , Wegner F. , Jensen I. et al., The Comorbidity and Co-Medication Profile of Patients With Progressive Supranuclear Palsy, Journal of Neurology. (2024) 271, no. 2, 782–793, 10.1007/s00415-023-12006-4. PMC10827866 37803149

[43] Bacchi S. , Chim I. , and Patel S. , Specificity and Sensitivity of Magnetic Resonance Imaging Findings in the Diagnosis of Progressive Supranuclear Palsy, Journal of Medical Imaging and Radiation Oncology. (2018) 62, no. 1, 21–31, 10.1111/1754-9485.12613. 28432758

[44] Spiegel C. , Marotta C. , Bertram K. , Vivash L. , and Harding I. H. , Brainstem and Cerebellar Radiological Findings in Progressive Supranuclear Palsy, Brain Communications. (2025) 7, no. 1, 10.1093/braincomms/fcaf051. PMC11829206 39958262

[45] Jecmenica L. , Kurz C. , Respondek G. et al., Copathology in Progressive Supranuclear Palsy: Does It Matter?, Movement Disorders: Official Journal of the Movement Disorder Society. (2020) 35, 984–993, 10.1002/mds.28011. 32125724

[46] Tepedino M. F. , Diana F. , Avallone A. R. et al., Investigating the Impact of White Matter Hyperintensities on Longitudinal Progression in Progressive Supranuclear Palsy, Neurological Sciences. (2025) 46, no. 12, 6933–6937, 10.1007/s10072-025-08547-3. 41107578

[47] Wen Y. , Zhou H. , Xia M. , Liu Q. , Quan H. , and Fang L. , Differentiating Progressive Supranuclear Palsy from Other Movement Disorders Using Transcranial Sonography: A Systematic Review and meta-analysis, Neurological Sciences. (2024) 45, no. 2, 455–465, 10.1007/s10072-023-07107-x. 37819487

[48] Bruno M. K. , Dhall R. , Duquette A. et al., A General Neurologist’s Practical Diagnostic Algorithm for Atypical Parkinsonian Disorders, Neurology. Clinical Practice. (2024) 14, no. 6, 10.1212/CPJ.0000000000200345. PMC11341009 39185098

[49] Krzosek P. , Madetko N. , Migda A. , Migda B. , Jaguś D. , and Alster P. , Differential Diagnosis of Rare Subtypes of Progressive Supranuclear Palsy and PSP-Like Syndromes—Infrequent Manifestations of the Most Common Form of Atypical Parkinsonism, Frontiers in Aging Neuroscience. (2022) 2022, 10.3389/fnagi.2022.804385. PMC8864174 35221993

[50] Murialdo A. , Marchese R. , Abbruzzese G. , Tabaton M. , Michelozzi G. , and Schiavoni S. , Neurosyphilis Presenting as Progressive Supranuclear Palsy, Movement Disorders. (2000) 15, no. 4, 730–731. 10.1002/1531-8257(200007)15:4<730::aid-mds1020>3.0.co;2-o 10928586

[51] Gaig C. , Graus F. , Compta Y. et al., Clinical Manifestations of the Anti-IgLON5 Disease, Neurology. (2017) 88, no. 18, 1736–1743, 10.1212/wnl.0000000000003887. PMC5409845 28381508

[52] Zhang Y. Z. , Ni Y. , Gao Y. N. et al., Anti-IgLON5 Disease: a Novel Topic Beyond Neuroimmunology, Neural Regeneration Research. (2023) 18, no. 5, 1017–1022, 10.4103/1673-5374.355742. PMC9827781 36254983

[53] Iyer J. M. , Gunzler D. , Lang A. E. et al., Concomitant Medications for Progressive Supranuclear Palsy: A Secondary Analysis of a Randomized Clinical Trial, JAMA Neurology. (2024) 81, no. 3, 295–297, 10.1001/jamaneurol.2023.5215. PMC10804278 38252447

[54] Rowe J. B. , Holland N. , and Rittman T. , Progressive Supranuclear Palsy: Diagnosis and Management, Practical Neurology. (2021) 21, no. 5, 376–383, 10.1136/practneurol-2020-002794. PMC8461411 34215700

[55] DeRosier F. , Hibbs C. , Alessi K. et al., Progressive Supranuclear Palsy: Neuropathology, Clinical Presentation, Diagnostic Challenges, Management, and Emerging Therapies, Disease-a-Month. (2024) 70, no. 8, 10.1016/j.disamonth.2024.101753. 38908985

[56] Dunning E. E. , Decourt B. , Zawia N. H. , Shill H. A. , and Sabbagh M. N. , Pharmacotherapies for the Treatment of Progressive Supranuclear Palsy: A Narrative Review, Neurology and Therapy. (2024) 13, no. 4, 975–1013, 10.1007/s40120-024-00614-9. PMC11263316 38743312

[57] Bluett B. , Pantelyat A. Y. , Litvan I. et al., Best Practices in the Clinical Management of Progressive Supranuclear Palsy and Corticobasal Syndrome: A Consensus Statement of the CurePSP Centers of Care, Frontiers in Neurology. (2021) 12, 10.3389/fneur.2021.694872. PMC8284317 34276544

[58] Ye L. , Sani S. S. , Veith Sanches L. et al., Acute Levodopa Challenge in Atypical Parkinsonism: Comprehensive Analysis of Individual Motor Responses, Brain Sciences. (2024) 14, no. 10, 10.3390/brainsci14100991. PMC11506334 39452005

[59] Slade S. C. , Finkelstein D. I. , McGinley J. L. , and Morris M. E. , Exercise and Physical Activity for People With Progressive Supranuclear Palsy: A Systematic Review. Clinical Rehabilitation, Clinical Rehabilitation. (2020) 34, no. 1, 23–33, 10.1177/0269215519877235. PMC6943961 31559853

[60] Pillas D. , Klein A. , Gasalla T. et al., The Burden of Progressive Supranuclear Palsy on Patients, Caregivers, and Healthcare Systems by PSP Phenotype: A Cross-Sectional Study, Frontiers in Neurology. (2022) 13, 10.3389/fneur.2022.821570. PMC9295700 35865639

[61] Street D. , Malpetti M. , Rittman T. et al., Clinical Progression of Progressive Supranuclear Palsy: Impact of Trials Bias and Phenotype Variants, Brain Communications. (2021) 3, no. 3, 10.1093/braincomms/fcab206. PMC8445397 34541533

[62] Campagnolo M. , Weis L. , Fogliano C. et al., Clinical, Cognitive, and Morphometric Profiles of Progressive Supranuclear Palsy Phenotypes, Journal of Neural Transmission. (2023) 130, no. 2, 97–109, 10.1007/s00702-023-02591-z. PMC9902314 36701008

[63] Swallow D. M. A. and Counsell C. E. , The Evolution of Diagnosis from Symptom Onset to Death in Progressive Supranuclear Palsy (PSP) and Corticobasal Degeneration (CBD) Compared to Parkinson’s Disease (PD), Journal of Neurology. (2023) 270, no. 7, 3464–3474, 10.1007/s00415-023-11629-x. PMC10266988 36971841

[64] Ali F. , Martin P. R. , Botha H. et al., Sensitivity and Specificity of Diagnostic Criteria for Progressive Supranuclear Palsy, Movement Disorders: Official Journal of the Movement Disorder Society. (2019) 34, no. 8, 1144–1153, 10.1002/mds.27619. PMC6688972 30726566

[65] Whitwell J. L. , Höglinger G. U. , Antonini A. et al., Radiological Biomarkers for Diagnosis in PSP: where are We and Where Do We Need to Be?, Movement Disorders: Official Journal of the Movement Disorder Society. (2017) 32, no. 7, 955–971, 10.1002/mds.27038. PMC5511762 28500751

[66] Tepedino M. F. , Diana F. , Abate F. et al., The Contribution of White Matter Changes to Clinical Phenotype in Progressive Supranuclear Palsy, Journal of Neurology. (2024) 271, no. 10, 6866–6875, 10.1007/s00415-024-12662-0. PMC11447107 39222284

[67] Williams D. R. , de Silva R. , Paviour D. C. et al., Characteristics of Two Distinct Clinical Phenotypes in Pathologically Proven Progressive Supranuclear Palsy: Richardson’s Syndrome and PSP-Parkinsonism, Brain: A Journal of Neurology. (2005) 128, no. Pt 6, 1247–1258, 10.1093/brain/awh488. 15788542

[68] Respondek G. , Stamelou M. , Kurz C. et al., The Phenotypic Spectrum of Progressive Supranuclear Palsy: a Retrospective Multicenter Study of 100 Definite Cases, Movement Disorders: Official Journal of the Movement Disorder Society. (2014) 29, no. 14, 1758–1766, 10.1002/mds.26054. 25370486

[69] Boxer A. L. , Yu J. T. , Golbe L. I. , Litvan I. , Lang A. E. , and Höglinger G. U. , Advances in Progressive Supranuclear Palsy: New Diagnostic Criteria, Biomarkers, and Therapeutic Approaches, The Lancet Neurology. (2017) 16, no. 7, 552–563, 10.1016/s1474-4422(17)30157-6. PMC5802400 28653647

[70] Dormann C. F. , Elith J. , Bacher S. et al., Collinearity: A Review of Methods to Deal with It and a Simulation Study Evaluating Their Performance, Ecography. (2013) 36, no. 1, 27–46, 10.1111/j.1600-0587.2012.07348.x.

[71] Vittinghoff E. and McCulloch C. E. , Relaxing the Rule of Ten Events per Variable in Logistic and Cox Regression, American Journal of Epidemiology. (2007) 165, no. 6, 710–718, 10.1093/aje/kwk052. 17182981

