# Supplementary material for: Clinical and Epidemiological Profile of Progressive Supranuclear Palsy in a Colombian Referral Center: A Retrospective Cohort Study
Source: Neurol Res Int. 2026 Oct 29;2026:9076106. doi: 10.1155/nri/9076106 (PMC13720349; doi:10.1155/nri/9076106)
Supplement: Supplementary file 1 [file NRI-2026-9076106-s001.docx]

**Supplementary table**

**Table S1. Sociodemographic and clinical characteristics according to progressive supranuclear palsy phenotype**

Percentages were calculated using available data for each variable; therefore, totals may not sum to 100% because of missing data.

|  |  | **PSP-RS (n = 50)** | **PSP-P (n = 49)** | **PSP-PI (n = 19)** | **PSP-F (n = 12)** | **PSP-SL (n = 3)** | **PSP-CBS (n = 4)** | **PSP-C (n = 3)** | **PSP-OM (n = 2)** | **PSP-PGF (n = 1)** | **Unclassified (n = 7)** |
| --- | --- | --- | --- | --- | --- | --- | --- | --- | --- | --- | --- |
| **Sex** | Women Men | 23 (46) 27 (54) | 19 (38.8) 30 (61.2) | 9 (47.4) 10 (52.6) | 7 (58.3)  5 (41.7) | 3 (100) 0 | 3 (75) 1 (25) | 1 (33.3) 2 (66.6) | 1 (50) 1 (50) | 0 1 (100) | 4 (57.1) 3 (42.9) |
| **Residence** | Metropolitan Area of Valle de Aburrá Outside the Metropolitan Area of Valle de Aburrá | 44 (88) 5 (10) | 41 (83.7) 8 (16.3) | 12 (63.2) 6 (12.2) | 9 (75) 3 (25) | 3 (100) 0 | 3 (75) 1 (25) | 3 (100) 0 | 2 (100) 0 | 1 (100) 0 | 4 (57.1) 3 (42.9) |
| **Age group, years** | <55 years | 1 (2) | 0 | 0 | 0 | 0 | 0 | 0 | 0 | 0 | 0 |
|  | 55-64 years | 3 (6) | 3 (6.1) | 2 (10.5) | 1 (8.3) | 0 | 1 (25) | 0 | 0 | 0 | 0 |
|  | 65-74 years | 13 (26) | 14 (28.6) | 5 (26.3) | 5 (41.7) | 0 | 0 | 0 | 0 | 0 | 2 (28.6) |
|  | 75-84 years | 21 (42) | 25 (51) | 5 (26.3) | 6 (50) | 3 (100) | 2 (50) | 3 (100) | 0 | 1 (100) | 4 (57.1) |
|  | 85-94 years | 10 (20) | 5 (10.2) | 7 (36.8) | 0 | 0 | 1 (25) | 0 | 1 (50) | 0 | 1 (14.3) |
|  | >=95 years | 2 (4) | 2 (4.1) | 0 | 0 | 0 | 0 | 0 | 1 (50) | 0 | 0 |
| **Age, years^a** | Mean ± SD [median (IQR)] | 77.5 ± 12.1 [79.5 (11.8)] | 77 ± 8.7 [77 (11)] | 78.7 ± 9.6 [79 (18.5)] | 73.6 ± 6.1 [74.5 (10.3)] | 81.7 ± 3.2 [83 (3)] | 78.5 ± 10.3 [81 (9)] | 77.7 ± 2.3 [79 (2)] | 92.5 ± 10.6 [92.5 (7.5)] | 77 | 77.3 ± 11.5 [83 (12)] |
| **Age at symptom onset, years^a** | Mean ± SD [median (IQR)] | 71.5 ± 8.3 [71 (11.75)] | 69.5 ± 9.5 [68 (13)] | 72.5 ± 9.15 [75 (14)] | 64.9 ± 5.9 [65 (5)] | 73.3 ± 6.6 [75 (6.5)] | 70 ± 10.8 [72.5 (14)] | 72 ± 5.3 [74 (5)] | 84.5 ± 5 [84.5 (4)] | 64 | 68.1 ± 9.8 [68 (11)] |
| **Time from symptom onset to diagnosis, years^a** | Mean ± SD [median (IQR)] | 2.9 ± 2.2 [2 (2.5)] | 3.9 ± 2.2 [4 (3)] | 2.83 ± 1.3 [3 (1)] | 3.6 ± 2.3 [3 (3.5)] | 4 ± 0 [4 (0)] | 3.3 ± 1.5 [3 (1.5)] | 3.5 ± 0.7 [3.5 (0.5)] | NC | NC | 7.3 ± 3 [8 (1.8)] |
| **Personal medical history** | Hypertension Type 2 diabetes mellitus Hypothyroidism Dyslipidemia >3 cardiovascular comorbidities Psychoaffective disorders Sleep disorders | 24 (48) 9 (18) 11 (22) 7 (14) 7 (14) 12 (24) 3 (6) | 21 (42.9) 7 (14.3) 9 (18.4) 7 (14.3) 4 (8.2) 3 (6.1) 3 (6.1) | 11 (57.9) 5 (26.3) 4 (21.1) 4 (21.1) 4 (21.1) 1 (5.3) 1 (5.3) | 6 (50) 4 (33.3) 4 (33.3) 2 (16.7) 2 (16.2) 1 (8.3) 0 | 1 (33.3) 0 2 (66.7) 0 0 0 0 | 4 (100) 2 (50) 0 0 1 (25) 1 (25) 0 | 1 (33.3) 1 (33.3) 0 1 (33.3) 0 1 (33.3) 1 (33.3) | 1 (50) 0 0 0 0 0 0 | 1 (100) 0 0 1 (100) 1 (100) 1 (100) 0 | 6 (85.7) 0 1 (14.3) 0 2 (28.6) 0 0 |
| **Presenting symptom** | Postural instability Akinesia or bradykinesia Cognitive dysfunction Oculomotor dysfunction Other | 19 (38) 19 (38) 6 (12) 2 (4) 4 (8) | 17 (34.7) 13 (26.5) 12 (24.5) 0 7 (14.3) | 10 (52.6) 3 (15.8) 4 (21.1) 1 (5.3) 1 (5.3) | 1 (8.3) 2 (16.7) 8 (66.7) 0 1 (8.3) | 0 2 (66.7) 1 (33.3) 0 0 | 2 (50) 1 (25) 0 1 (25) 0 | 3 (100) 0 0 0 0 | 0 0 0 2 (100) 0 | 0 0 1 (100) 0 0 | 3 (42.9) 0 1 (14.3) 0 3 (42.9) |
| **Time to diagnosis^b** | Days Months Years Missing data | 2 (4) 4 (8) 42 (84) 2 (4) | 3 (6.1) 2 (4.1) 43 (87.8) 1 (2) | 0 4 (33.3) 13 (68.4) 1 (5.3) | 0 2 (16.7) 9 (75) 1 (8.3) | 0 1 (33.3) 2 (66.7) 0 | 0 1 (25) 3 (75) 0 | 0 0 3 (100) 0 | 1 (50) 1 (50) 0 0 | 0 0 1 (100) 0 | 0 3 (42.9) 3 (42.9) 1 (14.3) |
| **Alternative diagnoses** | Parkinson's disease | 13 (26) | 14 () | 4 (33.3) | 1 (8.3) | 1 (33.3) | 0 | 0 | 0 | 0 | 0 |
|  | Alzheimer's disease | 1 (2) | 2 (4.1) | 1 (5.3) | 2 (16.7) | 0 | 1 (25) | 0 | 0 | 0 | 0 |
|  | Essential tremor | 1 (2) | 1 (2) | 0 | 0 | 0 | 1 (25) | 0 | 0 | 0 | 0 |
|  | Unspecified tremor | 0 | 1 (2) | 0 | 1 (8.3) | 0 | 0 | 0 | 0 | 0 | 0 |
|  | Dementia with Lewy bodies | 1 (2) | 1 (2) | 0 | 0 | 0 | 0 | 0 | 0 | 0 | 0 |
|  | Unspecified ataxia | 0 | 0 | 0 | 0 | 0 | 1 (25) | 1 (33.3) | 0 | 0 | 0 |
|  | Multiple system atrophy | 0 | 2 (4.1) | 0 | 0 | 0 | 0 | 0 | 0 | 0 | 0 |
|  | Myelopathy | 0 | 1 (2) | 0 | 0 | 0 | 0 | 0 | 0 | 0 | 0 |
|  | Primary lateral sclerosis | 0 | 0 | 0 | 0 | 0 | 0 | 0 | 0 | 0 | 1 (14.3) |
|  | Sequelae of cervical myelopathy | 1 (2) | 0 | 0 | 0 | 0 | 0 | 0 | 0 | 0 | 0 |
|  | Normal-pressure hydrocephalus | 0 | 0 | 0 | 1 (8.3) | 0 | 0 | 0 | 0 | 0 | 0 |
|  | Anterograde amnesia | 0 | 0 | 1 (5.3) | 0 | 0 | 0 | 0 | 0 | 0 | 0 |
|  | Transient ischemic attack | 0 | 0 | 0 | 0 | 0 | 0 | 0 | 0 | 0 | 1 (14.3) |
|  | Multifactorial dementia | 0 | 1 (2) | 0 | 0 | 0 | 0 | 0 | 0 | 0 | 0 |
|  | Corticobasal degeneration | 0 | 0 | 0 | 1 (8.3) | 0 | 0 | 0 | 0 | 0 | 0 |
|  | Wernicke's disease | 0 | 0 | 0 | 0 | 0 | 0 | 0 | 0 | 1 (100) | 0 |
|  | Lacunar infarction in the basal ganglia | 1 (2) | 0 | 0 | 0 | 0 | 0 | 0 | 0 | 0 | 0 |
| **VDRL testing performed** | Yes | 38 (76) | 35 (71.4) | 11 (57.9) | 10 (83.3) | 3 (100) | 4 (100) | 2 (66.7) | 2 (100) | 1 (100) | 5 (71.4) |
| **CSF analysis performed** | Yes | 11 (22) | 35 (71.4) | 0 | 3 (25) | 0 | 2 (50) | 0 | 1 (50) | 0 | 0 |
| **Neuroimaging studies** | MRI Non-contrast CT Transcranial sonography (TCS) Contrast-enhanced CT Magnetic resonance angiography (MRA) | 42 (84) 18 (36) 7 (14) 2 (4) 2 (4) | 39 (79.6) 12 (24.5) 5 (10.2) 1 (2) 0 | 14 (73.7) 2 (10.5) 1 (5.3) 0 0 | 10 (83.3) 6 (50) 1 (8.3) 1 (8.3) 0 | 3 (100) 1 (33.3) 0 0 0 | 4 (100) 1 (25) 1 (25) 0 0 | 3 (100) 1 (33.3) 0 0 0 | 2 (100) 0 0 0 0 | 1 (100) 0 0 0 0 | 7 (100) 4 (57.1) 2 (28.6) 0 0 |
| **Neuroimaging findings** | "Mickey Mouse sign" "Penguin silhouette sign" "Morning glory sign" Hypometabolism on functional imaging Lenticular hyperechogenicity and ventricular enlargement White matter microangiopathy Frontotemporal and/or cerebellar atrophy Midbrain atrophy | 2 (4) 0 2 (4) 0 1 (2) 14 (28) 12 (24) 22 (44) | 2 (4.1) 1 (2) 2 (4.1) 0 1 (2) 17 (34.7) 4 (8.2) 22 (44.9) | 0 0 0 0 0 4 (21.1) 3 (15.8) 3 (15.8) | 1 (8.3) 0 0 0 0 5 (41.7) 4 (33.3) 3 (25) | 0 0 0 0 0 0 1 (33.3) 1 (33.3) | 0 0 0 0 0 0 0 2 (50) | 1 (33.3) 0 0 0 0 0 1 (33.3) 3 (100) | 0 0 0 0 0 0 0 0 | 0 0 0 0 0 1 (100) 0 0 | 0 0 0 0 0 3 (42.9) 2 (28.6) 0 |
| **Type of therapy** | Monotherapy Polytherapy Missing data | 3 (6) 46 (92) 1 (2) | 9 (18.4) 40 (81.6) 0 | 1 (5.3) 18 (94.7) 0 | 3(25) 8(66.7) 1 (8.3) | 2(66.7) 1(33.3) 0 | 0 4(100) 0 | 0 3(100) 0 | 2 (100) 0 0 | 0 1 (100) 0 | 1 (14.3) 5 (71.4) 1 (14.3) |
| **Medications used** | Levodopa/carbidopa SSRIs Amantadine Botulinum toxin Zolpidem | 48 (96) 42 (84) 2 (4) 1 (2) 3 (6) | 46 (93.9) 35 (71.4) 4 (8.2) 2 (4.1) 4 (8.2) | 19 (100) 18 (94.7) 0 0 0 | 8 (66.7) 7 (58.3) 1 (8.3) 2 (16.7) 1 (8.3) | 3 (100) 1 (33.3) 0 0 0 | 4 (100) 3 (75) 1 (25) 1 (25) 0 | 3 (100) 3 (100) 0 0 0 | 0 0 0 1 (50) 1 (50) | 1 (100) 1 (100) 0 0 0 | 7 (100) 6 (85.7) 2 (28.6) 0 0 |
| **Non-pharmacological management** | Psychotherapy Neurorehabilitation Psychotherapy plus neurorehabilitation Bioenergetic therapy Missing data | 3 (6) 19 (38) 3 (6) 0 25 (50) | 1 (2) 15 (30.6) 1 (2) 0 30 (61.2) | 1 (5.3) 9 (47.4) 0 0 9 (47.4) | 3 (25) 1 (8.3) 1 (8.3) 0 7 (58.3) | 0 1 (33.3) 0 1 (33.3) 1 (33.3) | 1 (25) 2 (50) 0 0 1 (25) | 0 0 0 0 3 (100) | 0 1 (50) 0 0 1 (50) | 0 0 0 0 1 (100) | 0 4 (57.1) 0 0 5 (71.4) |
| **Documented initial levodopa response** | Yes | 11 (22) | 17 (34.7) | 2 (10.5) | 1 (8.3) | 0 | 0 | 2 (66.7) | 0 | 0 | 1 (14.3) |

**Notes.** Data are presented as n (%) unless otherwise indicated. **^a** Mean ± standard deviation [median (interquartile range)]. **^b** Time from symptom onset to diagnosis. NC indicates that the value could not be calculated because of insufficient data.

**Abbreviations.** CSF, cerebrospinal fluid; CT, computed tomography; MRA, magnetic resonance angiography; MRI, magnetic resonance imaging; PSP, progressive supranuclear palsy; PSP-C, PSP with predominant cerebellar ataxia; PSP-CBS, PSP with corticobasal syndrome; PSP-F, PSP with predominant frontal presentation; PSP-OM, PSP with predominant oculomotor dysfunction; PSP-P, PSP-parkinsonism; PSP-PGF, PSP with progressive gait freezing; PSP-PI, PSP with predominant postural instability; PSP-RS, PSP-Richardson syndrome; PSP-SL, PSP with predominant speech/language disorder; SSRIs, selective serotonin reuptake inhibitors; TCS, transcranial sonography; VDRL, Venereal Disease Research Laboratory test.
